# Effects of neonatal hypothyroidism on testicular development and undifferentiated spermatogonia in prepubertal rats

**DOI:** 10.1111/andr.70116

**Published:** 2025-09-12

**Authors:** Daisuke Matsumoto, Kentaro Mizuno, Hidenori Nishio, Hideyuki Kamisawa, Takuya Sakata, Taiki Kato, Akihiro Nakane, Satoshi Kurokawa, Tetsuji Maruyama, Yutaro Hayashi, Takahiro Yasui

**Affiliations:** ^1^ Department of Nephro‐urology Nagoya City University Graduate School of Medical Sciences Nagoya Japan; ^2^ Department of Pediatric Urology Nagoya City University Graduate School of Medical Sciences Nagoya Japan; ^3^ Department of Urology Anjo Kosei Hospital Anjo Japan; ^4^ Department of Urology Tokyo Adventist Hospital & Clinic Tokyo Japan; ^5^ Department of Urology Gamagori City Hospital Gamagori Japan; ^6^ Department of Urology Nagoya City University East Medical Center Nagoya Japan

**Keywords:** hypothyroidism, neonatal, prepuberty, spermatogenesis, spermatogonial stem cells, undifferentiated spermatogonia

## Abstract

**Background:**

Thyroid hormones play a key role in testicular development, particularly in the regulation of Sertoli cell proliferation and differentiation. While congenital hypothyroidism is common and treatable, the effects of thyroid hormone insufficiency on early testicular development during the neonatal period remain unclear.

**Objectives:**

This study investigated the effects of transient and continuous hypothyroidism during the neonatal and prepubertal periods on testicular development, focusing on spermatogonial stem cell dynamics through histological and germ cell marker analyses.

**Materials and methods:**

We established two neonatal rat models using 6‐n‐propyl‐2‐thiouracil: a continuous hypothyroidism model and a transient neonatal hypothyroidism model. 6‐n‐Propyl‐2‐thiouracil was administered to lactating dams at concentrations of 0.001%, 0.01%, and 0.03%. Male offspring were evaluated on postnatal days 7, 10, and 20 for serum hormone levels, body and testicular growth, and immunohistochemical markers (GFRA1, DDX4, SOX9, and Ki‐67).

**Results:**

The transient hypothyroidism model successfully induced transient hypothyroidism without systemic growth impairment. Serum thyroxine and thyroid‐stimulating hormone levels were normalized by day 20. GFRA1‐positive undifferentiated germ cells consistently increased in all 6‐n‐propyl‐2‐thiouracil groups on days 7 and 20. Co‐expression with Ki‐67 indicated cell proliferation. The formation of seminiferous tubule lumen was reduced in a dose‐dependent manner.

**Discussion:**

Transient neonatal hypothyroidism increases the number of undifferentiated germ cells, potentially including spermatogonial stem cells. The transient hypothyroidism model minimizes systemic effects and allows the observation of testis‐specific responses to thyroid disruption.

**Conclusion:**

This study demonstrated that even low‐dose transient hypothyroidism during the neonatal period enhances the population of undifferentiated germ cells, potentially including spermatogonial stem cells. The transient hypothyroidism model offers a physiologically relevant and minimally invasive platform to explore how early thyroid hormone imbalances influence germ cell population establishment during a critical window of testicular development, potentially reflecting the clinical scenarios of treated congenital hypothyroidism.

## 1 INTRODUCTION

Spermatogenesis is a complex and continuous process that produces spermatozoa and progresses continuously after puberty.^1^ Spermatogonial stem cells (SSCs) are the origin of spermatozoa, which differentiate from gonocytes during infancy. Research on humans has reported a correlation between the number of spermatogonia including SSCs in infancy and future sperm count, highlighting the critical importance of spermatogonia in infancy from a fertility perspective.^2^ However, the regulatory mechanisms controlling the differentiation and proliferation of SSCs from gonocytes remain unclear.

Our previous research focused on the differentiation of SSCs through studies on cryptorchidism, a congenital disorder characterized by undescended testes.^3^, ^4^, ^5^

Although the exact mechanisms underlying SSC reduction in cryptorchidism remain unclear, this decrease is likely attributed to endocrine abnormalities in certain cases.^6^ In human research, we have shown that markers such as follicle‐stimulating hormone (FSH), anti‐Müllerian hormone, and inhibin B can be used to predict SSC numbers in the testicular tissue of cryptorchidism,^7^ suggesting a link between hormonal abnormalities and SSC reduction.

Among the various hormonal influences on testicular development, thyroid hormones are known to play a key role in metabolism and modulate Sertoli cell proliferation and differentiation.^8^ Thyroid dysfunction, including both hypothyroidism and hyperthyroidism, has been shown to alter spermatogenesis in rodents and humans. In rodents, the administration of 6‐n‐propyl‐2‐thiouracil (PTU), which induces reversible hypothyroidism, extends the mitotic period of Sertoli cells by approximately 2 weeks, significantly increasing testis size, Sertoli cell numbers per testis, and sperm production in adult animals.^9^, ^10^, ^11^ Conversely, hyperthyroidism induced by triiodothyronine (T3) accelerates Sertoli cell differentiation and maturation, reducing the number of Sertoli cells per testis and sperm production.^12^, ^13^

Congenital hypothyroidism (CH) is a common neonatal endocrine disorder with an estimated incidence of one in 2000–10,000 live births.^14^ Early treatment usually ensures normal pubertal development and gonadal functions. Nevertheless, little is known about how early testicular development is affected in individuals treated for CH during the neonatal period, and the implications for the initial establishment of the germ cell population remain unclear.

To investigate the effects of hypothyroidism during early life on testicular development—particularly in undifferentiated spermatogonia, potentially including SSCs—we developed two neonatal rat models: one with continuous hypothyroidism (cHT) and another with transient hypothyroidism (tHT) limited to the neonatal period. Specifically, we clarified the primary objective of this study, which was to investigate the effects of transient and cHT during the neonatal and prepubertal periods on testicular development, including SSC dynamics, by analyzing testicular histology and germ cell markers at key developmental stages.

## 2 MATERIALS AND METHODS

### 2.1 Animals

Pregnant Sprague–Dawley (SD) rats (8 weeks old) were obtained from Japan SLC, Inc. The rats were maintained in a controlled environment with a 12‐h light/12‐h dark cycle and ad libitum access to water. The rats were administered a standard laboratory diet. Each rat was housed in a separate cage. All experimental procedures were approved by the Animal Care Committee of Nagoya City University Graduate School of Medical Sciences, Nagoya, Japan (approval number: H28_M65).

### 2.2 Continuous hypothyroid model

We used PTU (Sigma Chemical) to induce hypothyroidism in male rat offspring. Pregnant SD rats were orally administered PTU (concentrations of 0.001%, 0.01%, and 0.03%) mixed with water after delivery. PTU was transferred to the newborns via milk to develop a hypothyroid rat model. The control group was administered tap water and maintained under typical experimental conditions. In the continuous hypothyroid (cHT) model, PTU was administered continuously from birth until postnatal day 20 throughout the experimental period.

### 2.3 Transient hypothyroid model

Similar to the cHT model, the groups were divided and PTU was administered; however, in the transient hypothyroid (tHT) model, PTU was administered only from birth until 7 days postnatally. Subsequently, the three PTU‐treated groups and the control group were administered tap water.

### 2.4 Animal tissue preparation

The cHT and tHT model rats were euthanized by exsanguination via the inferior vena cava after isoflurane inhalation. Blood samples and testes were collected on postnatal days 7, 10, and 20 (*n* = 5). The experimental procedures were performed in the morning hours. Blood samples were processed to obtain serum, which was frozen and used to measure the hormone levels. The collected testicular tissues were fixed in 4% paraformaldehyde, embedded in paraffin, and used for histological evaluation.

### 2.5 Serum hormone level assays

Serum thyroxine (T4), triiodothyronine (T3), thyroid‐stimulating hormone (TSH), FSH, and luteinizing hormone (LH) levels were measured using a Luminex MAGPIX system (Merck). To measure serum T4, T3, and TSH levels, the MILLIPLEX Rat Thyroid Magnetic Bead Panel (#RTHYMAG‐30K) was used, and for FSH and LH, the MILLIPLEX Rat Pituitary Magnetic Bead Panel (#RPTMAG‐86K) was used. T3 and T4 levels were measured as the total concentrations of T3 and T4 in the blood. Standards, quality controls, and samples were evaluated in duplicate, and the average value was used. Serum T4, T3, and TSH levels were compared among the control, 0.001% PTU, 0.01% PTU, and 0.03% PTU groups. Serum FSH and LH levels were compared between the control and 0.01% PTU‐treated groups.

### 2.6 Antibodies for immunohistochemistry analysis

DDX4, GFRA1, and SOX9 were used as markers for germ cells, undifferentiated spermatogonia, including SSCs, and Sertoli cells, respectively. Ki‐67 was used as a marker of cell proliferation for fluorescence immunostaining. The following primary antibodies were used in this study: (a) anti‐DDX4 (1:5000; ab13840; Abcam), (b) anti‐GFRA1 (1:100; AF560; R&D Systems), (c) anti‐SOX9 (1:300; #82630; Cell Signaling Technology), and (d) anti‐Ki‐67 (1:1000; ab15580; Abcam).

### 2.7 Testicular histopathology and immunohistochemistry

Testicular samples collected on postnatal days 7 and 20 were fixed in 4% paraformaldehyde, embedded in paraffin, and sectioned to obtain slices with a thickness of 5 µm. Sections were deparaffinized in Hemo‐D (Falma) (thrice, 10 min each) and 100% ethanol (thrice, 10 min each), rehydrated in a graded ethanol series (90%, 80%, and 70%; 2 min each), and treated with MilliQ‐H_2_O (5 min). The sections were heated at 95°C for 40 min in 10 mM sodium citrate dehydrate solution (pH 6) in a Decloaking Chamber NxGen (Biocare Medical), cooled at room temperature (20°C–25°C) for 30 min, and treated with methanol containing 3% H_2_O_2_ to prevent endogenous peroxidase activity from interfering with detection. To block non‐specific staining, the sections were treated with 5% skim milk diluted in phosphate‐buffered saline with Tween 20 for 1 h and incubated overnight at 4°C with antibodies targeting DDX4, GFRA1, and SOX9. For negative control staining, testicular sections were processed following the same immunohistochemical procedure, except that the primary antibodies for DDX4, GFRA1, or SOX9 were omitted (Figure 5Q‒X). The sections were then incubated at room temperature with a biotin‐labeled antibody (VECTASTAIN Elite ABC kit) and an avidin‐biotinylated peroxidase complex (VECTASTAIN Elite ABC kit) for 30 min each. After incubation with 3,3′‐diaminobenzidine tetrahydrochloride solution at 21°C, the sections were counterstained with hematoxylin. The sections were dehydrated in a graded ethanol series (70%, 80%, 90%, and 100%; 2 min each), cleared with xylene, and mounted. For immunostaining, digital images were acquired using a BZ‐X800 (Keyence) and analyzed using a BZ‐X800 analyzer.

### 2.8 Measurement of the seminiferous tubule diameter in the tHT model

We performed hematoxylin and eosin (H&E) staining of testicular tissues derived from the control group and the three groups treated with PTU on postnatal days 7 and 20. We measured the short diameter (µm) of the cross‐sections of the seminiferous tubules. For each group (*n* = 5), approximately 50 seminiferous tubules per testis sample, which were as close to perfectly circular as possible, were selected and counted, and average values were calculated.

### 2.9 Measurement of the average number of positive cells per cross‐section of seminiferous tubules in the tHT model

We performed immunostaining of testicular tissues from the control and three PTU‐treated groups on postnatal day 7 using antibodies targeting three proteins (DDX4, GFRA1, and SOX9) to measure the number of positive cells per cross‐section of the seminiferous tubules. Additionally, on postnatal day 20, we measured the number of positive cells per cross‐section of the seminiferous tubules using antibodies targeting two proteins (GFRA1 and SOX9). Approximately 100 nearly circular seminiferous tubules per testis specimen were selected for counting in each group (*n* = 5), and the average number was calculated. All evaluations were performed by a single examiner in a non‐blinded manner.

### 2.10 Measurement of the average number of DDX4‐positive cells in contact with the basal membrane per cross‐section of seminiferous tubules in the tHT model

We performed immunostaining of testicular tissues from the control and PTU‐treated groups on postnatal day 20 using an anti‐DDX4 antibody. We measured the number of positive cells in contact with the basal membrane of the seminiferous tubules in cross‐sections and calculated the number of positive cells per seminiferous tubule. For each group (*n* = 5), approximately 50 seminiferous tubules per testis sample, which were as close to perfectly circular as possible, were selected and counted, and average values were calculated.

### 2.11 Double immunofluorescence staining for GFRA1 and Ki‑67

To assess the proliferative status of GFRA1‑positive cells, double immunofluorescence staining was performed on testicular sections obtained from the tHT model (0.01% PTU) on postnatal day 7. Paraffin‐embedded testicular sections (5 µm thick) were deparaffinized, rehydrated, and subjected to antigen retrieval using 10 mM sodium citrate buffer (pH 6.0) at 95°C for 40 min. After cooling to room temperature (20°C–25°C) and blocking with 5% skim milk diluted in phosphate‐buffered saline with Tween 20 for 1 h, the sections were incubated overnight at 4°C with primary antibodies against GFRA1 (1:100; AF560; R&D Systems) and Ki‑67 (1:1000; ab15580; Abcam). On the following day, the sections were first incubated at room temperature for 2 h with a donkey anti‐goat Alexa Fluor 647 secondary antibody (1:2000; Invitrogen) for GFRA1 detection. After washing, DAPI and a goat anti‑rabbit Alexa Fluor 546 secondary antibody (1:2000; Life Technologies) were added for Ki‑67 detection, and the sections were incubated for 1 h at room temperature. The slides were then mounted with an antifade mounting medium and visualized using a fluorescence microscope (BZ‑X800, Keyence). Representative images were selected from the five biological replicates.

### 2.12 Statistical analysis

All statistical analyses were performed using EZR,^15^ which is a statistical software that extends the functions of R and R Commanders. For each group (*n* = 5), individual mean values per animal were calculated, and group comparisons were performed based on the distribution of these values. Data are presented as box plots indicating the median and interquartile range. Differences between the two groups were evaluated using the Mann–Whitney *U* test. Statistical significance was set at *p* < 0.05.

## 3 RESULTS

### 3.1 Testicular development and hormonal status in the cHT model

**FIGURE 1:**
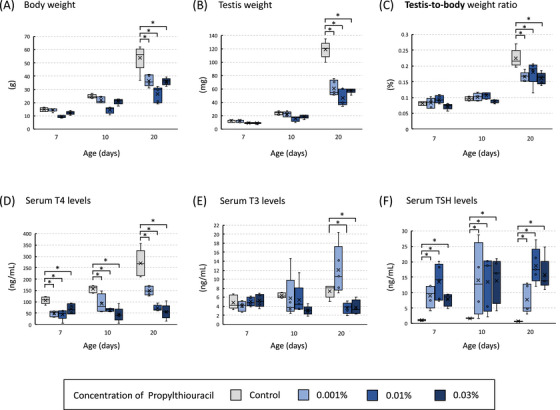
Body weight (A), testis weight (B), testis‐to‐body weight ratio (C), and serum levels of thyroxine (T4) (D), triiodothyronine (T3) (E), and thyroid‐stimulating hormone (TSH) (F) on postnatal days 7, 10, and 20 in continuous hypothyroidism (cHT) model rats. Three 6‐n‐propyl‐2‐thiouracil (PTU)‐treated groups exhibited a significant decrease in body weight, testis weight, and testis‐to‐body weight ratio on postnatal day 20 compared to the control group. Serum T4 levels were significantly decreased in the three PTU‐treated groups on postnatal days 7, 10, and 20 days. A decrease in T3 levels was observed in the 0.01% PTU and 0.03% PTU groups on day 20. TSH levels showed a significant increase in all three PTU‐treated groups throughout the study period. The asterisk indicates a significant difference compared to the control group (^*^ *p* < 0.05).

In the cHT model, the three PTU‐treated groups exhibited a significant decrease in body and testis weights on postnatal day 20 compared with to the control group (Figure 1A,B). The testis‐to‐body weight ratio was lower in the PTU‐treated groups (Figure 1C). Additionally, mortality was observed in the 0.03% PTU group, in which two of the 51 juvenile rats died before postnatal day 20. No deaths occurred in the 0.01% PTU, 0.001% PTU, or control group. Serum T4 levels markedly decreased in the three PTU‐treated groups on postnatal days 7, 10, and 20 (Figure 1D). Regarding serum T3 levels, a decrease was observed in the 0.01% and 0.03% PTU groups on day 20 (Figure 1E). Serum TSH levels increased significantly in the three PTU‐treated groups throughout the study period (Figure 1F).

### 3.2 Testicular development and hormonal status in the tHT model

**FIGURE 2:**
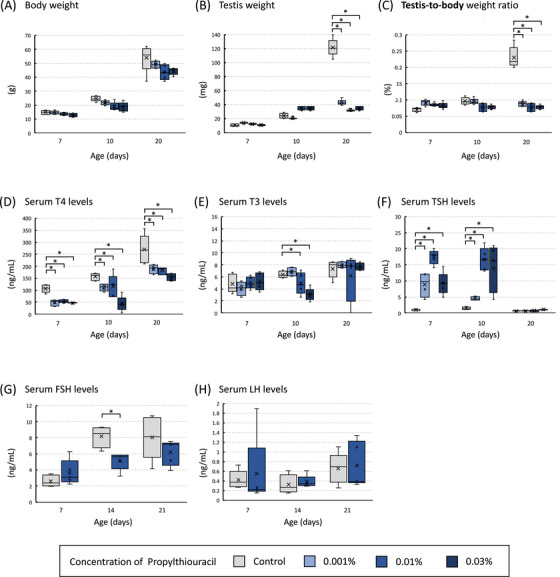
Body weight (A), testis weight (B), testis‐to‐body weight ratio (C), and serum levels of thyroxine (T4) (D), triiodothyronine (T3) (E), and thyroid‐stimulating hormone (TSH) (F) on postnatal days 7, 10, and 20, and follicle‐stimulating hormone (FSH) (G) and luteinizing hormone (LH) (H) levels on postnatal days 7, 14, and 20 in transient hypothyroidism (tHT) model rats. No significant differences were observed in body weight, testis weight, or testis‐to‐body weight ratio between the control and 6‐n‐propyl‐2‐thiouracil (PTU)‐treated groups. Compared to the control group, the 0.001% PTU, 0.01% PTU, and 0.03% PTU groups exhibited a significant decrease in serum T4 levels throughout the study period. For serum T3 levels, a significant decrease was observed in the 0.01% PTU and 0.03% PTU groups on postnatal day 10, whereas no significant difference was noted on postnatal day 20. Serum TSH levels were significantly higher in all PTU‐treated groups than in the control group on postnatal days 7 and 10; however, by postnatal day 20, all groups showed similar levels. For serum FSH, a significant decrease was observed in the 0.01% PTU group compared to the control group on postnatal day 10. No significant differences were observed in serum LH levels between the two groups. The asterisk indicates a significant difference compared to the control group (^*^ *p* < 0.05).

In the tHT model, body weight remained comparable between the control and PTU‐treated groups at all examined time points (postnatal days 7, 10, and 20) (Figure 2A). Testis weight and the testis‐to‐body weight ratio did not differ significantly on days 7 and 10; however, both values were significantly lower in the PTU‐treated groups than in the control group on day 20 (Figure 2B,C). Compared with the control group, the 0.001% PTU, 0.01% PTU, and 0.03% PTU groups exhibited a significant decrease in serum T4 levels on postnatal days 7, 10, and 20 (Figure 2D). A significant decrease in serum T3 levels was observed in the 0.01% PTU and 0.03% PTU groups on postnatal day 10, whereas no significant difference was noted on postnatal day 20 (Figure 2E). Serum TSH levels were significantly higher in all PTU‐treated groups than in the control group on postnatal days 7 and 10. However, by postnatal day 20, all groups showed similar levels (Figure 2F). A significant decrease in serum FSH levels was observed in the 0.01% PTU group compared to the control group on postnatal day 10 (Figure 2G). No significant differences were observed in serum LH levels between the two groups (Figure 2H).

### 3.3 Histopathological changes in the testes on postnatal day 7

**FIGURE 3:**
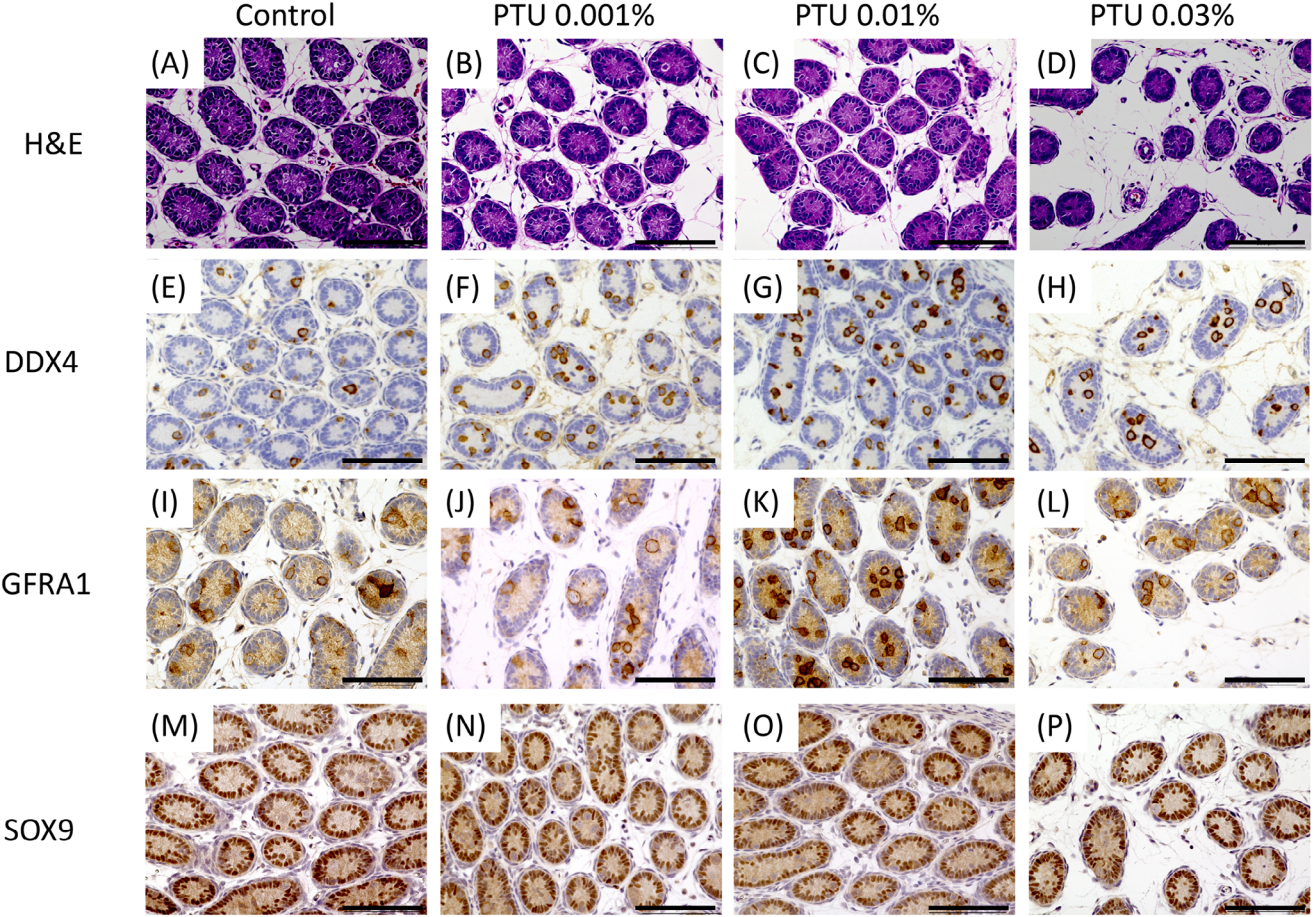
Hematoxylin and eosin staining and images of immunostaining images of testes with anti‐DDX4, anti‐GFRA1, and anti‐SOX9 antibodies in the control group and 0.001% 6‐n‐propyl‐2‐thiouracil (PTU), 0.01% PTU, and 0.03% PTU groups on postnatal day 7 in both the continuous hypothyroidism (cHT) and transient hypothyroidism (tHT) models. Within the seminiferous tubules, germ cells and Sertoli cells were similarly observed across all groups; however, as the PTU concentration increased, the seminiferous tubules appeared narrower, and the surrounding interstitial areas appeared wider (A‒D). Immunostaining with the anti‐DDX4 (E‒H) and anti‐GFRA1 (I‒L) antibodies showed positive staining in germ cells, which exhibited large, round nuclei located within or at the periphery of the seminiferous tubules. Compared to the control group, the three PTU‐treated groups had a higher number of positive cells per cross‐section of the seminiferous tubules. Staining with the anti‐SOX9 antibody (M‒P) revealed positive Sertoli cells with spindle‐shaped nuclei at the periphery of the seminiferous tubules, showing a similar staining pattern across all groups. Scale bar = 100 µm.

We conducted a histological analysis of the testes from all four groups on postnatal day 7 in both the cHT and tHT models across all four groups. H&E staining revealed no significant changes in the structure of the seminiferous tubules; however, alterations in the diameter of the tubules and surrounding interstitial areas were observed. Immunostaining with antibodies targeting DDX4, GFRA1, and SOX9 indicated that the average number of positive cells for DDX4 and GFRA1 per seminiferous tubule appeared to increase in the three PTU‐treated groups compared to that in the control group, whereas no differences were observed among the groups for SOX9 (Figure 3).

### 3.4 Histopathological changes in the testes on postnatal day 20

Histological analysis of the testes was performed on postnatal day 20 in both the cHT and tHT models.

**FIGURE 4:**
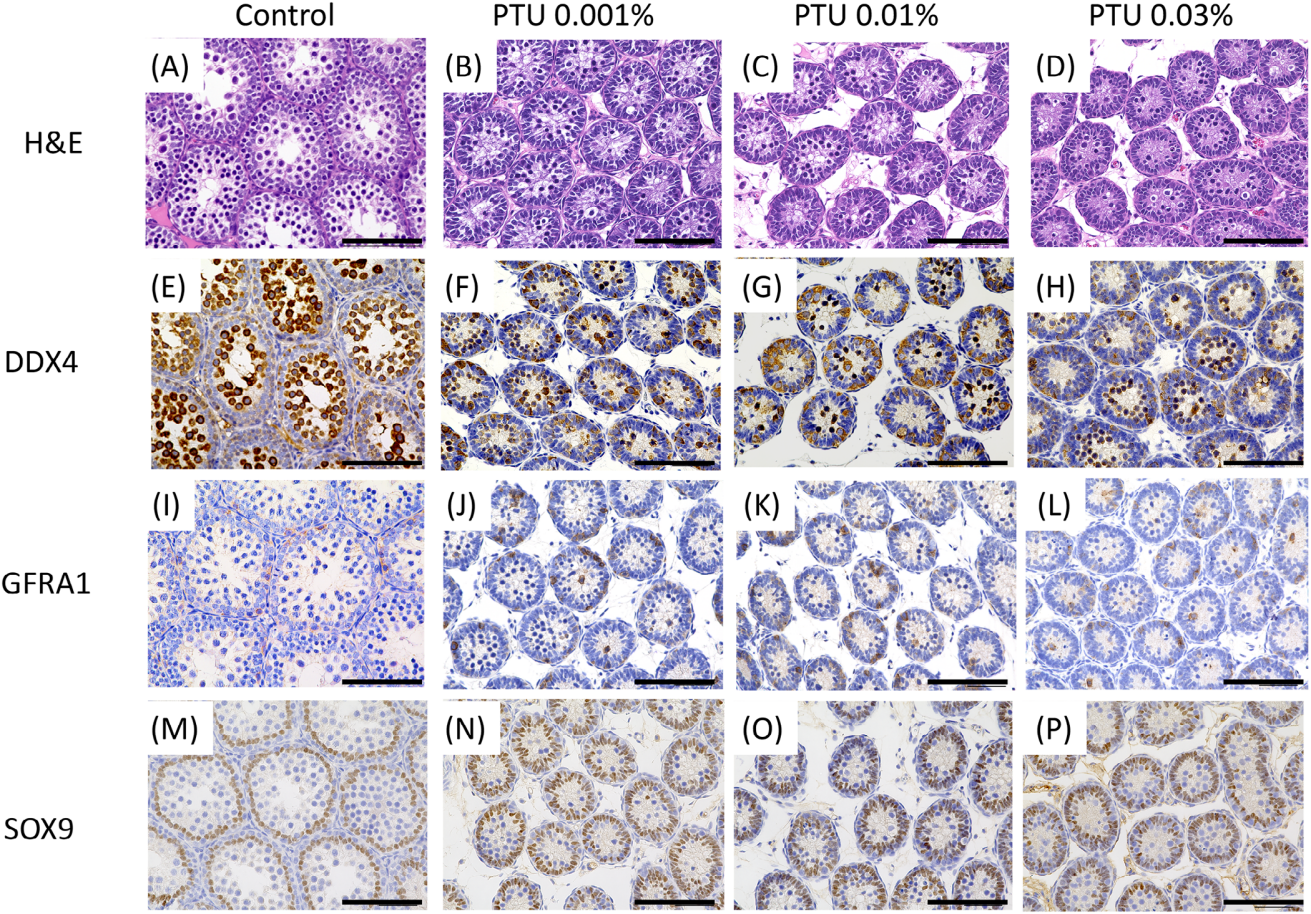
Hematoxylin and eosin staining and immunostaining images of testes with anti‐DDX4, anti‐GFRA1, and anti‐SOX9 antibodies in the control, 0.001% 6‐n‐propyl‐2‐thiouracil (PTU), 0.01% PTU, and 0.03% PTU groups on postnatal day 20 in continuous hypothyroidism (cHT) model rats. In the control group, the seminiferous tubules exhibited clear lumen formation, whereas no lumen formation was observed in any of the PTU‐treated groups (A‒D). These groups also showed markedly reduced seminiferous tubule diameters and expanded interstitial areas compared to the control group. Immunostaining with the anti‐DDX4 antibody (E‒H) revealed positive germ cells in all groups, but the number of DDX4‐positive cells per cross‐section was consistently lower in the PTU‐treated groups. Although GFRA1‐positive (I‒L) and SOX9‐positive (M‒P) cells were present in the PTU‐treated groups, the significant morphological differences in seminiferous tubules and developmental stages made direct quantitative comparisons with the control group inappropriate. Scale bar = 100 µm.

In the cHT model (Figure 4), H&E staining showed clear lumen formation within seminiferous tubules in the control group, whereas no tubule lumen formation was observed in any of the three PTU‐treated groups. These groups also exhibited markedly reduced seminiferous tubule diameters and expanded interstitial areas compared with those in the control group. Immunostaining with an anti‐DDX4 antibody revealed positive cells in all groups, but the number of DDX4‐positive cells was consistently lower in the PTU‐treated groups than in the control group. Although GFRA1‐ and SOX9‐positive cells were also detected in the PTU‐treated groups, the obvious differences in tubule diameter and developmental stage made quantitative comparison with the control group inappropriate.

**FIGURE 5:**
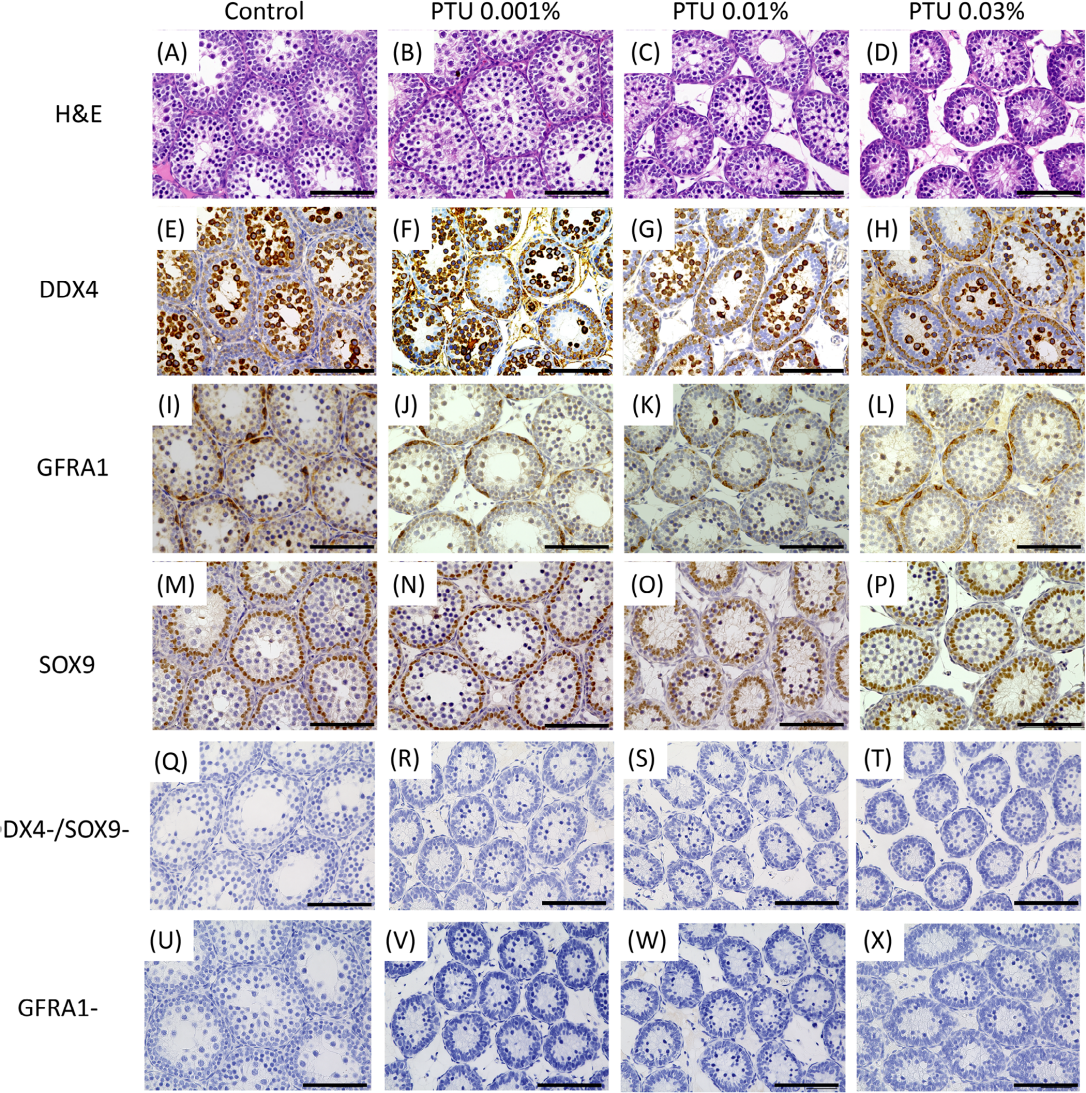
Hematoxylin and eosin staining and immunostaining images of testes with anti‐DDX4, anti‐GFRA1, and anti‐SOX9 antibodies in the control, 0.001% 6‐n‐propyl‐2‐thiouracil (PTU), 0.01% PTU, and 0.03% PTU groups on postnatal day 20 in transient hypothyroidism (tHT) model rats. In all groups, the development of the seminiferous tubule lumen structure was observed; however, as the PTU concentration increased, the proliferation and differentiation of spermatocytes into mature stages of the cell life cycle were not observed, and the seminiferous tubules were narrower, with wider surrounding interstitial areas (A‒D). Immunostaining with the anti‐DDX4 antibody (E‒H) revealed positive germ cells with large, round, or semicircular nuclei located in the marginal region adjacent to the basement membrane of the seminiferous tubules. The number of positive cells per cross‐section of the seminiferous tubules was higher in the three PTU‐treated groups than in the control group. Immunostaining with the anti‐GFRA1 antibody (I‒L) showed positive cells in the marginal region of the seminiferous tubules, with a higher number of positive cells per cross‐section in the three PTU‐treated groups. Staining with the anti‐SOX9 antibody (M‒P) revealed positive Sertoli cells with spindle‐shaped nuclei at the periphery of the seminiferous tubules, displaying similar staining patterns in all groups. Negative control for DDX4, GFRA1, and SOX9 immunostaining in the testis of the tHT model on postnatal day 20 (Q‒X). Scale bar = 100 µm.

In contrast, the tHT model (Figure 5) showed seminiferous tubule lumen formation in all groups. Variations in tubule diameter and interstitial morphology were observed on postnatal day 7. Immunostaining with an anti‐DDX4 antibody demonstrated an increased number of positive cells along the basement membrane in the PTU‐treated groups compared to the control group. Similarly, the number of GFRA1‐positive cells per seminiferous tubule was higher in the PTU‐treated groups, whereas no significant differences in SOX9‐positive cell numbers were observed among the groups.

### 3.5 Diameter of seminiferous tubules in the tHT model

**FIGURE 6:**
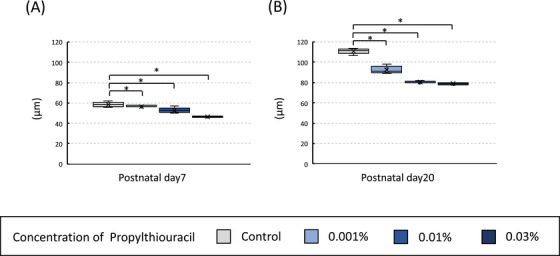
Comparison of short diameter of the seminiferous tubules among four groups on postnatal days 7 (A) and 20 (B) in the transient hypothyroidism (tHT) model. In all cases, the seminiferous tubule diameter was smaller in the 6‐n‐propyl‐2‐thiouracil (PTU)‐treated groups than in the control group, showing a dose‐dependent trend. The asterisk indicates a significant difference compared to the control group (^*^ *p* < 0.05).

The short diameter of the seminiferous tubules was measured on postnatal days 7 (Figure 6A) and 20 (Figure 6B). In all cases, the PTU‐treated groups showed smaller diameters than the control group, with a dose‐dependent trend.

### 3.6 Comparison of positive cell counts for immunostaining (DDX4, GFRA1, and SOX9) among four groups on postnatal days 7 and 20 in the tHT model

**FIGURE 7:**
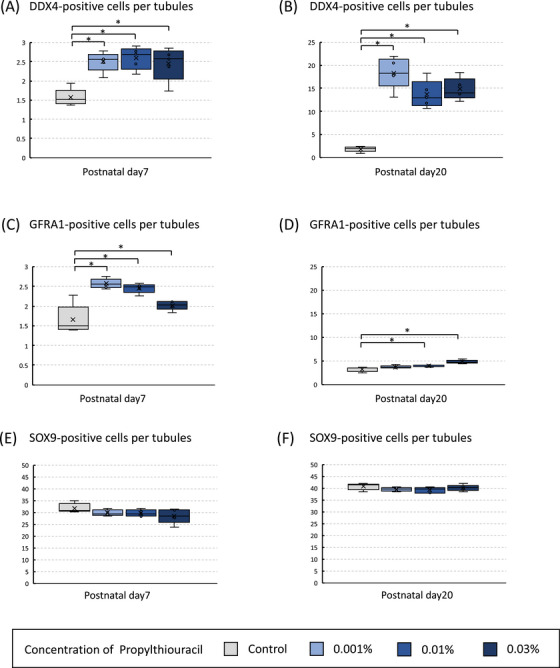
Comparison of positive cell counts for immunostaining (DDX4, GFRA1, and SOX9) among the four groups on postnatal days 7 and 20 in the transient hypothyroidism (tHT) model. On postnatal day 7, the three 6‐n‐propyl‐2‐thiouracil (PTU)‐treated groups exhibited a comparable increase in the number of DDX4‐positive cells per cross‐section of the seminiferous tubules compared to the control group (A and C). However, no significant differences were observed among the four groups for SOX9 (E). Furthermore, on postnatal day 20, the three PTU‐treated groups showed a significant increase in the average number of DDX4‐positive cells in the peripheral region per cross‐section of the seminiferous tubules compared to the control group (B). Additionally, the 0.01% PTU and 0.03% PTU groups exhibited a significant increase in the number of GFRA1‐positive cells per cross‐section compared to that in the control group (D). As with postnatal day 7, no significant differences were observed among the four groups for SOX9 (F). An asterisk indicates a significant difference from the control group (^*^ *p* < 0.05).

In the tHT model, positive cell counts were measured on postnatal days 7 and 20 and compared among the four groups by immunostaining with antibodies targeting three proteins: DDX4, GFRA1, and SOX9. On postnatal day 7, the three PTU‐treated groups exhibited a comparable increase in the number of DDX4‐ and GFRA1‐positive cells per cross‐section of the seminiferous tubules compared to the control group (Figure 7A,C); however, no significant differences were observed for SOX9 (Figure 7E). Furthermore, on postnatal day 20, the three PTU‐treated groups showed a significant increase in the average number of DDX4‐positive cells in the peripheral region per cross‐section of the seminiferous tubules compared to the control group (Figure 7B). Additionally, the 0.01% PTU and 0.03% PTU groups exhibited a significant increase in the number of GFRA1‐positive cells per cross‐section compared to the control group (Figure 7D). As with postnatal day 7, no significant differences were observed among the four groups for SOX9 (Figure 7F).

### 3.7 Double immunofluorescence staining for GFRA1 and Ki‐67

**FIGURE 8:**
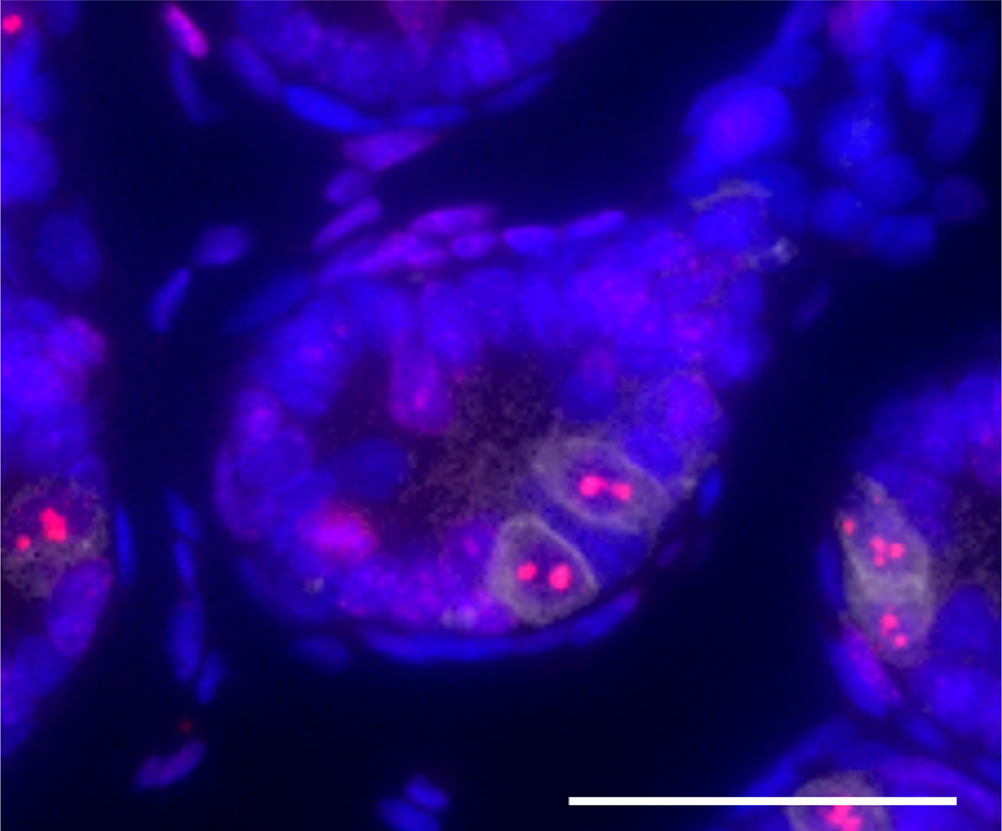
GFRA1/Ki‐67 double immunofluorescence staining in the transient hypothyroidism (tHT) model (0.01% 6‐n‐propyl‐2‐thiouracil [PTU]) on postnatal day 7. Two GFRA1‐positive cells (green) were observed along the periphery of the seminiferous tubule located at the center of the image. Ki‐67‐positive nucleoli (red) were present within the nuclei of these cells. Scale bar = 50 µm.

In the testes of the tHT model (0.01% PTU) on postnatal day 7, two GFRA1‐positive cells were identified along the periphery of a seminiferous tubule located at the center of the image. Ki‐67‐positive nucleoli were detected within the nuclei of these cells (Figure 8).

## 4 DISCUSSION

This study utilized two experimental models, cHT and tHT, to investigate the effects of neonatal hypothyroidism on testicular tissue. We employed the anti‐thyroid drug PTU, which inhibits the conversion of T4 to T3, thereby reducing thyroid hormone levels.^16^, ^17^ PTU has been used in experimental animals to induce hypothyroidism and study spermatogenesis.^18^ In the cHT model used in this study, hypothyroidism was successfully induced. However, the cHT model, which involves sustained hypothyroidism throughout the neonatal and prepubertal periods, induces marked systemic effects, including significant reductions in body weight and organ development. These generalized impairments obscure the interpretation of testis‐specific findings, making it difficult to determine whether the observed histological and cellular changes are a direct result of thyroid dysfunction or secondary to overall developmental delay. Therefore, the cHT model may not be suitable for evaluating the specific relationship between thyroid function and testicular development, particularly regarding SSC dynamics. Conversely, in the tHT model, a decrease in T4 levels and a transient increase in TSH levels because of negative feedback were observed until postnatal day 10. TSH levels returned to control levels by day 20, and the difference in T4 levels decreased. These results suggest the establishment of a neonatal transient hypothyroidism model. Importantly, there were no significant differences in body weight between the PTU‐treated groups and the control groups, indicating that the tHT model exerted minimal systemic effects. This supports the validity of this model for specifically examining the effects of transient hypothyroidism on testicular development. To our knowledge, this is the first study to confirm a transient hypothyroid state during the critical prepubertal period until postnatal day 20 based on blood data. Clinically, this model may closely reflect CH that is treated during the neonatal to prepubertal period and was designed to examine how transient neonatal hypothyroidism influences germ cell populations and early testicular development during this sensitive developmental window. Although the small body size of neonatal rats generally limits the volume of blood that can be collected, the use of the MAGPIX system enabled the accurate and simultaneous measurement of multiple serum hormones, allowing us to obtain a detailed endocrine profile.

On postnatal days 7 and 20, GFRA1‐positive cells, indicative of undifferentiated spermatogonia, consistently increased in all PTU‐treated groups compared to the controls, whereas Sertoli cell counts remained unchanged. Furthermore, the proportion of tubules exhibiting lumen formation, which is indicative of seminiferous tubule maturation, was lower in the PTU groups than in the control group and tended to decrease with higher PTU doses.

Recent studies have revealed the effects of thyroid hormones on spermatogenesis. Thyroid hormone acts on TRα1, a thyroid hormone receptor expressed in Sertoli cell nuclei, inhibiting Sertoli cell proliferation and promoting their functional maturation in prepubertal testes, whereas hypothyroidism is thought to stimulate immature Sertoli cell proliferation.^19^ Sertoli cells are essential somatic elements in testicular differentiation, proliferating robustly prenatally and continuing until approximately 3 weeks postnatally in rats.^20^ Subsequently, Sertoli cell numbers stabilize throughout the animal's lifetime.^21^ By extending the proliferative period, hypothyroidism may increase the number of Sertoli cells in adulthood.^22^ Various studies on PTU‐induced transient hypothyroidism in prepubertal rats have reported increased adult Sertoli cell counts, functional sperm counts, and germ cell numbers.^18^, ^23^ In contrast, hyperthyroidism induced by T3 administration accelerates Sertoli cell differentiation and maturation, thereby reducing adult Sertoli cell count and sperm production.^12^, ^24^ However, few studies have assessed testicular tissue, focusing on SSCs within the critical prepubertal period, particularly before 2 weeks of age. Auharek and de França^25^ reported no significant difference in Sertoli cell counts among the control, hypothyroidism, and hyperthyroidism groups on postnatal day 5; however, by day 10, Sertoli cell counts increased in the hypothyroid group and decreased in the hyperthyroid group. The authors also noted corresponding changes in germ cell counts, with hypothyroid groups showing increased counts and the hyperthyroid groups showing decreased counts; these trends persisted even after day 100.^25^

This study evaluated testicular tissue on postnatal days 7 and 20, and no significant differences were observed in Sertoli cell counts between the control and PTU‐treated groups. Given the younger age of the rats compared to those in previous studies, it is plausible that this period was insufficient to reveal differences in Sertoli cell numbers. However, germ cell counts significantly increased in the PTU‐treated groups on days 7 and 20, with an increase in DDX4‐ and GFRA1‐positive cells. DDX4‐positive cells are expressed across all differentiation stages of germ cells, from undifferentiated germ cells to round spermatids.^26^ GFRA1‐positive cells are thought to be enriched in the SSC population and are frequently used as surrogate markers for SSCs.^27^ In addition to GFRA1, several other molecular markers for SSCs have been identified, including ID4, FGFR3, UTF1, and KIT.^28^ Although the timing of marker expression differs, the precise functions of the cells expressing these markers remain incompletely characterized. Due to the lack of a specific marker for SSCs, marker expression alone is insufficient to definitively identify functional SSCs, and spermatogonial transplantation remains the only definitive method for identifying SSCs.^29^ Therefore, because SSCs cannot be definitively identified based on GFRA1 expression alone, an increase in undifferentiated spermatogonia—potentially including SSCs—was observed in the PTU‐treated groups up to postnatal day 20.

In neonatal rodents, stem cell properties are observed in a small population of undifferentiated spermatogonia termed Aund cells, which are categorized as Asingle, Apaired, and Aaligned.^30^ Aligned chains differentiate into A1 spermatogonia, followed by various mitotic divisions into A2, A3, A4, intermediate, and type B spermatogonia, ultimately forming leptotene spermatocytes that initiate the first meiotic division.^30^ GFRA1 is primarily expressed in both single and Apaired cells.^31^, ^32^ While germ cell categorization traditionally relies on morphological criteria outlined by Vergouwen et al.^33^ and Chiarini‐Garcia and Russell,^34^ immunohistochemistry has enabled classification using protein markers. Although morphological studies have assessed changes in germ cell numbers in neonatal transient hypothyroidism models, to our knowledge, this is the first study to evaluate germ cell count variations using functional protein markers. All three hypothyroid groups showed increased numbers of GFRA1‐positive germ cells, including SSCs. In the 0.01% PTU group on postnatal day 7, double immunofluorescence staining revealed that some of the GFRA1‐positive spermatogonia were also Ki‐67‐positive, indicating that a portion of these cells was actively proliferating.^35^ This observation may represent the initial stage of a previously reported phenomenon in which transient hypothyroidism during the prepubertal period leads to an increase in sperm count and germ cell numbers in adulthood. These findings on neonatal transient hypothyroidism‐induced germ cell count increases before puberty represent an important discovery with potential implications for future studies on SSC.

In this study, three PTU dose groups (0.001%, 0.01%, and 0.03%) were used to examine the serum hormone levels and testicular tissue changes. Higher PTU doses corresponded to greater reductions in T4 levels. Even the remarkably low 0.001% PTU concentration significantly reduced serum T4 levels compared to the controls. Serum TSH levels also increased in all PTU groups, indicating successful induction of hypothyroidism. T3 levels showed no significant differences on day 7, but by day 10, only the 0.01% PTU and 0.03% PTU groups exhibited decreases, which resolved by day 20. The absence of T3 reduction on day 7 is likely because of compensatory mechanisms; T3 is derived peripherally from T4, and early stage T4 reduction often triggers an increase in T4‐to‐T3 conversion.^36^ Moreover, slower T3 degradation, as T3 levels decrease, further mitigates the systemic T3 reduction.^37^ Although the direct effect of PTU on the testis requires consideration, it is unlikely to be significant based on two key factors: the established safety of PTU in clinical hyperthyroidism treatment without reported testicular toxicity and multiple studies indicating no significant histological changes when T4 supplementation normalizes testicular tissue in PTU‐induced hypothyroid models.^38^

Most previous studies on PTU‐induced hypothyroidism have used PTU concentrations between 0.01% and 0.5%.^39^, ^40^ This study uniquely demonstrated that even a remarkably low concentration of 0.001% PTU can induce hypothyroidism, with thyroid function normalizing more swiftly. Increased undifferentiated germ cell counts were observed in the 0.001% PTU group, an effect that has not been previously reported. Although transient hypothyroidism may positively influence spermatogenesis by increasing the future sperm count, it temporarily impairs systemic metabolism. Therefore, lower PTU concentrations may be preferable for SSC research and applications, rendering the findings of this study significant.

Recent research on FSH has indicated its differential roles during puberty: it promotes Sertoli cell proliferation prepubertally and enhances their differentiation in adulthood.^41^ In our study, a transient FSH reduction was observed on postnatal day 14 in the PTU‐treated groups. This delayed transient decrease in FSH levels following transient hypothyroidism may indicate a feedback response to hypothyroidism‐induced Sertoli cell proliferation; however, further investigation is necessary to clarify this mechanism.

This study had some limitations. Although we focused on the histological effects of hypothyroidism on neonatal spermatogenesis, the molecular mechanisms underlying these findings remain unclear and warrant further research.

In conclusion, this study demonstrated that even a remarkably low concentration of PTU and a short, transient hypothyroid state from the neonatal to prepubertal period led to an increase in undifferentiated spermatogonia, a population that likely includes SSCs. The tHT model, which induces minimal systemic effects by restricting hypothyroidism to the neonatal period, may offer a reliable experimental system for investigating the specific impact of thyroid hormone disruption on SSC maintenance and testicular development. Notably, this model may also clinically reflect CH diagnosed and treated during the neonatal to prepubertal period, highlighting its potential utility in exploring the mechanisms by which early thyroid dysfunction and its correction influence reproductive development.

## AUTHOR CONTRIBUTIONS

Daisuke Matsumoto conceived the study under the supervision of Kentaro Mizuno. Daisuke Matsumoto performed most of the study, assisted by Taiki Kato and Kentaro Mizuno. Daisuke Matsumoto, Hidenori Nishio, Hideyuki Kamisawa, and Takuya Sakata collected data. Satoshi Kurokawa, Akihiro Nakane, and Tetsuji Maruyama contributed to the data analysis. Kentaro Mizuno assisted with statistical examinations. Takahiro Yasui and Yutaro Hayashi revised the manuscript, and all the authors approved the final version.

## CONFLICT OF INTEREST STATEMENT

The authors declare they have no conflicts of interest.

## Data Availability

The datasets generated and/or analyzed during the current study are available from the corresponding author upon reasonable request.
